# Suicide attempt and suicide in refugees in Sweden – a nationwide population-based cohort study

**DOI:** 10.1017/S0033291719003167

**Published:** 2021-01

**Authors:** Ridwanul Amin, Magnus Helgesson, Bo Runeson, Petter Tinghög, Lars Mehlum, Ping Qin, Emily A. Holmes, Ellenor Mittendorfer-Rutz

**Affiliations:** 1 Division of Insurance Medicine, Department of Clinical Neuroscience, Karolinska Institutet, SE-171 77 Stockholm, Sweden; 2 Department of Clinical Neuroscience, Centre for Psychiatry Research, S.t Göran's Hospital, Karolinska Institutet, Stockholm County Council, SE-112 81 Stockholm, Sweden; 3 Swedish Red Cross University College, Hälsovägen 11, SE-141 57 Huddinge, Sweden; 4 National Centre for Suicide Research and Prevention, University of Oslo, Sognsvannsveien 21, NO-0374 Oslo, Norway; 5 Division of Psychology, Department of Clinical Neuroscience, Karolinska Institutet, SE-171 77 Stockholm, Sweden; 6 Department of Psychology, Uppsala University, Von Kraemers allé 1A and 1C, SE-752 37 Uppsala, Sweden

**Keywords:** Labour market marginalisation, migration, refugees, sick leave, suicide attempt, suicide

## Abstract

**Background:**

Despite a reported high rate of mental disorders in refugees, scientific knowledge on their risk of suicide attempt and suicide is scarce. We aimed to investigate (1) the risk of suicide attempt and suicide in refugees in Sweden, according to their country of birth, compared with Swedish-born individuals and (2) to what extent time period effects, socio-demographics, labour market marginalisation (LMM) and morbidity explain these associations.

**Methods:**

Three cohorts comprising the entire population of Sweden, 16–64 years at 31 December 1999, 2004 and 2009 (around 5 million each, of which 3.3–5.0% refugees), were followed for 4 years each through register linkage. Additionally, the 2004 cohort was followed for 9 years, to allow analyses by refugees' country of birth. Crude and multivariate hazard ratios (HRs) with 95% confidence intervals (CIs) were computed. The multivariate models were adjusted for socio-demographic, LMM and morbidity factors.

**Results:**

In multivariate analyses, HRs regarding suicide attempt and suicide in refugees, compared with Swedish-born, ranged from 0.38–1.25 and 0.16–1.20 according to country of birth, respectively. Results were either non-significant or showed lower risks for refugees. Exceptions were refugees from Iran (HR 1.25; 95% CI 1.14–1.41) for suicide attempt. The risk for suicide attempt in refugees compared with the Swedish-born diminished slightly across time periods.

**Conclusions:**

Refugees seem to be protected from suicide attempt and suicide relative to Swedish-born, which calls for more studies to disentangle underlying risk and protective factors.

## Background

Due to the increasing global migration in recent years, the demography of Sweden has changed, as it has in several other European countries (Swedish Migration Agency, 2018). Sweden received historically high numbers of individuals seeking asylum in recent years, granting the highest number of asylums per capita in the European Union in 2016 (Eurostat, 2017). In 2017, 18.5% of all people living in Sweden were foreign-born (Statistics Sweden, 2018).

Refugees have been reported to have a high prevalence of common mental disorders, particularly post-traumatic stress disorder (Bogic *et al*., 2015; Tinghög *et al*., 2017), which in turn can lead to suicidal behaviour (suicide attempt and suicide) (Ferrada-Noli *et al*., 1998; van Heeringen, 2012). Suicidal behaviour has a multifactorial aetiology and can be conceptualised by a stress-diathesis model which implies that acquired susceptibility arising from heredity interacts with stressful life events, takes a long-term toll on mental health and subsequently, increases the risk of suicidal behaviour (van Heeringen, 2012). For refugees, the process of migration, accompanied by traumatic experiences of war and torture, hazardous journeys, harsh circumstances in refugee camps and separation from family members (Tinghög, 2009), can be considered as such stressful life events. Experience of traumatic events may also lead to the development of common mental disorders. Moreover, post-migration difficulties including psychosocial acculturation problems and ethnical discrimination may adversely affect mental health and increase the risk of suicidal behaviour (Berry *et al*., 2002; Tinghög, 2009).

Despite this, there is a considerable lack of studies on suicide risk among refugees in an entire host country, and to the best of our knowledge there are no such studies on suicide attempt. One study in Denmark reported a significantly lower risk of suicide for male but not female refugees compared with the Danish-born population (Norredam *et al*., 2013). However, this study did not investigate differences in risk of suicide in refugees according to country of birth. Such knowledge is crucial as refugees form a heterogeneous group not only in terms of their ethnicity but also regarding the diverse nature of their traumatic experiences (Tinghög, 2009). Here, the culture, the dominating religion, the stigma associated with suicidal behaviour prevailing in the country of birth and the heterogeneity of traumas experienced by the refugees may have an influence on the individual's attitudes towards suicidal behaviour and, if a person acts on suicidal ideation (Tinghög, 2009; Lawrence *et al*., 2016).

Research on the risk of suicidal behaviour in refugees should not only consider information on the country of birth, but also on potential time period effects in the association between refugee status and subsequent suicidal behaviour. Here, time period effects might be related to temporal changes in e.g. the design of national social insurance and migration policies and/or the healthcare system in the host country. Several such changes have occurred during the last 20 years in Sweden. For example, mental healthcare reforms in the 1990s aimed at deinstitutionalisation and promoted more community support and outpatient care for individuals with severe mental disorders, which could be important in our study context (Bulow *et al*., 2002). Moreover, changes towards stricter regulations of the social insurance system in 2008 in Sweden might have affected the degree of labour market marginalisation (LMM) (Wang *et al*., 2016). Such changes, in turn, might be related to differential processes regarding acculturation, social integration and healthcare utilisation of refugees, which might be reflected in changes in the risk of suicidal behaviour over time (Tinghög, 2009). Furthermore, changes in the composition of the refugee population in terms of country of birth over the last decades in Sweden (Swedish Migration Agency, 2018) could also contribute to time period effects. Another important factor that needs attention in relation to possible time period effects is a potential change in rates of LMM over time, as immigrants (including refugees) are reported to be more marginalised at the labour market than individuals in the host country are (Helgesson *et al*., 2017). To the best of our knowledge, no previous study has examined to what extent time period effects explain the risk of suicidal behaviour in refugees.

### Aims

We aimed to investigate (1) the risk of suicide attempt and suicide in refugees in Sweden, according to their country of birth, compared with that of the Swedish-born population and (2) to what extent time period effects, socio-demographics, previous LMM and morbidity explain these associations.

## Material and methods:

### Design and study population

In this nationwide study, three cohorts comprising all individuals with a refugee or Swedish-born background, aged 16–64 years and residing in Sweden on the 31 of December in 1999 (*n* = 4 993 691), 2004 (*n* = 5 083 618) and 2009 (*n* = 5 171 135) were followed prospectively with regard to inpatient healthcare due to first incident suicide attempt as well as death due to suicide by means of register linkage for 4 years each. This means a follow-up from 1 January 2000, 2005 and 2010 to 31 December 2003, 2008 and 2013, respectively. The ‘2004’ cohort was also followed for a total of 9 years (until 31 December 2013) to enhance statistical power in the analyses regarding the risk of suicide attempt and suicide in refugees from specific countries of birth. The study population mentioned above excludes individuals with missing or erroneous data regarding their reason for residence (3.7% in the initial 1999 and 2009 cohort, 3.9% in the initial 2004 cohort).

### Data sources

Longitudinal data for each individual were available up to 31 December 2013 through register linkages from the following sources:

(1) Statistics Sweden: LISA database (Longitudinal integration database for health insurance and labour market studies (Statistics Sweden, 2016)) contains personal data on socio-demographic factors and LMM such as age, sex, country of birth, educational level, family situation, type of residential area, number of annual net days with sickness absence, disability pension and number of annual days with unemployment from 1990 onwards; STATIV database (Longitudinal database for integration studies) includes data on reason for residence (e.g. refugee) from 1997.
(2) National Board of Health and Welfare: National patient register with data on date and diagnosis of inpatient and specialised outpatient healthcare starting from 1987 and 2001, respectively and Cause of death register (date and cause of death from 1960 and onwards) (Brooke *et al*., 2017).

### Refugees and the Swedish-born population

In this study, all individuals grouped under the headings ‘refugee’, ‘in need of protection’ and ‘humanitarian grounds’ as their reason for residence at the Swedish Migration Agency were identified as refugees (Statistics Sweden, 2011). Refugees were defined according to the Geneva Convention on Refugees – a person who is outside his/her country of citizenship because of well-founded grounds for fear of persecution, and is unable to obtain sanctuary from the home country or, owing to such fear, is unwilling to avail himself of the protection of that country (UNHCR, 2018). A sensitivity analysis was performed to confirm the comparability of the estimates for the outcome measures, i.e. including or excluding individuals who were granted residence permits due to ‘in need of protection’ and on ‘humanitarian grounds’ as refugees. These estimates (data not shown) were similar to our results. All individuals with Sweden as their country of birth were defined as ‘Swedish-born’ in this study. Individuals with missing or erroneous data regarding their reason for residence were excluded from the initial study population (3.7% in the 1999 and 2009 cohort, 3.9% in the 2004 cohort).

### Exposure measures

The exposed groups in the analyses were refugees in total and according to their country of birth. The following countries generated the largest number of refugees to Sweden: Eritrea, Ethiopia, Somalia, Afghanistan, Iran, Iraq, Syria, Chile and the former Yugoslavia. Therefore, refugees born in these countries were grouped separately for additional analyses with specific country of birth (Tables 2 and 3). The reference group comprised the Swedish-born population, i.e. with Sweden as a country of birth.

### Covariates

A. *Socio-demographic factors*: sex, age, educational level, family situation and type of residential area; B. *LMM factors*: days with full-time unemployment, net days with sickness absence (e.g. 4 days on 25% or 2 days on 50% sickness absence gives one net day of sickness absence), granted disability pension and C. *Morbidity factors*: history of suicide attempt; history of inpatient or specialised outpatient healthcare (main or side diagnosis of specific somatic or psychiatric disorders). Diagnostic information was based on the codes of the International Classification of Diseases version 10 (ICD-10). Specific diagnoses are mentioned in online Supplementary Table S1. The socio-demographic and LMM factors were measured at the end and during the baseline year of each cohort (1999, 2004 and 2009), respectively. History of suicide attempt and of inpatient healthcare was measured for 5 years preceding the start of the follow-up (1995–1999, 2000–2004 and 2005–2009 for the respective cohort). For the 2004 cohort, in the analyses with 9 years of follow-up, specialised outpatient healthcare was also included and both inpatient and specialised outpatient healthcare were measured for 4 years preceding the start of the follow-up (2001–2004). Inclusion of the specialised outpatient healthcare data improved the coverage of the morbidity factors in the 2004 cohort with 9 years of follow-up. However, these data were only available from 2001 and onwards. Therefore, to ensure comparability, they were not included for adjustment in the 4-year-follow-up cohorts (1999, 2004 and 2009 cohorts). Missing values for a covariate were categorised in separate categories. Table 1 shows the categorisation of the covariates.

**Table 1.** Descriptive statistics of socio-demographic, LMM and morbidity characteristics of individuals aged 16–64 years with Swedish-born or refugee backgrounds^a^ in Sweden in 1999, 2004 and 2009 (*N* = 4 993 691, 5 083 618 and 5 171 135 respectively)

	1999 cohort, *n* (%)		2004 cohort, *n* (%)		2009 cohort, *n* (%)	
Characteristics	Swedish-born	Refugees	Swedish-born	Refugees	Swedish-born	Refugees
All	4 826 870 (96.7)	166 821 (3.3)	4 886 677 (96.1)	196 941 (3.9)	4 912 487 (95.0)	258 648 (5.0)
Socio-demographic factors (1999, 2004, 2009)						
Sex						
Women	2 363 765 (49.0)	69 674 (41.8)	2 390 145 (48.9)	83 512 (42.4)	2 401 482 (48.9)	109 292 (42.3)
Men	2 463 105 (51.0)	97 147 (58.2)	2 496 532 (51.1)	113 429 (57.6)	2 511 003 (51.1)	149 356 (57.7)
Age (years)						
16–24	822 837 (17.0)	31 526 (18.9)	847 767 (17.3)	41 996 (21.3)	960 363 (19.5)	46 820 (18.1)
25–34	1 065 778 (22.1)	44 137 (26.5)	970 084 (19.9)	39 923 (20.3)	888 315 (18.1)	57 667 (22.3)
35–44	1 001 522 (20.7)	57 389 (34.4)	1 049 949 (21.5)	61 761 (31.4)	1 048 786 (21.3)	62 365 (24.1)
45–54	1 074 450 (22.3)	24 991 (15.0)	983 745 (20.1)	40 046 (20.3)	984 653 (20.0)	64 437 (24.9)
55–64	862 283 (17.9)	8778 (5.3)	1 035 132 (21.2)	13 215 (6.7)	1 030 368 (21.0)	27 359 (10.6)
Educational level (years)						
Compulsory school (0–9)	1 251 922 (25.9)	53 686 (32.2)	1 037 420 (21.2)	56 880 (28.9)	935 490 (19.0)	71 143 (27.5)
High school (10–12)	2 273 241 (47.1)	61 240 (36.7)	2 323 813 (47.6)	78 611 (39.9)	2 326 662 (47.4)	100 602 (38.9)
College or university (>12)	1 273 092 (26.4)	37 105 (22.2)	1 495 059 (30.6)	49 534 (25.2)	1 617 798 (32.9)	72 976 (28.2)
Missing	28 615 (0.6)	14 790 (8.9)	30 385 (0.6)	11 916 (6.1)	32 535 (0.7)	13 927 (5.4)
Family situation						
Married/living with partner without children living at home	756 416 (15.7)	12 230 (7.3)	746 964 (15.3)	14 930 (7.6)	681 151 (13.9)	22 950 (8.9)
Married/living with partner with children living at home	1 633 863 (33.8)	74 804 (44.8)	1 604 384 (32.8)	84 941 (43.1)	1 614 819 (32.9)	109 051 (42.2)
Single/divorced/separated/widowed without children living at home	1 762 574 (36.5)	49 939 (29.9)	1 806 342 (37.0)	60 430 (30.7)	1 814 683 (36.9)	88 010 (34.0)
Single/divorced/separated/widowed with children living at home	278 890 (5.8)	13 607 (8.2)	292 521 (6.0)	16 090 (8.2)	293 537 (6.0)	21 524 (8.3)
Children (⩽20 years old) living at home	395 119 (8.2)	16 238 (9.7)	436 461 (8.9)	20 544 (10.4)	508 293 (10.3)	17 111 (6.6)
Missing	2 (0)	2 (0)	5 (0)	6 (0)	2 (0)	2 (0)
Type of residential area^b^						
Big cities	1 625 821 (33.7)	83 834 (50.3)	1 670 359 (34.2)	95 622 (48.6)	1 719 179 (35.0)	123 692 (47.8)
Medium-sized cities	1 732 079 (35.9)	57 312 (34.4)	1 766 126 (36.1)	70 680 (35.9)	1 775 365 (36.1)	94 405 (36.5)
Small cities/villages	1 468 970 (30.4)	25 675 (15.4)	1 450 192 (29.7)	30 639 (15.6)	1 417 941 (28.9)	40 551 (15.7)
LMM at baseline (1999, 2004, 2009)						
Unemployed, 1–180 days^c^	541 054 (11.2)	44 440 (26.6)	460 316 (9.4)	36 362 (18.5)	380 626 (7.7)	41 087 (15.9)
Unemployed, >180 days^c^	144 335 (3.0)	17 871 (10.7)	125 275 (2.6)	13 950 (7.1)	106 733 (2.2)	23 109 (8.9)
Sickness absence, 1–90 net days^d^	416 676 (8.6)	9693 (5.8)	344 858 (7.1)	12 679 (6.4)	301 814 (6.1)	13 676 (5.3)
Sickness absence, >90 net days^d^	187 541 (3.9)	4859 (2.9)	247 068 (5.1)	11 398 (5.8)	102 966 (2.1)	6810 (2.6)
Disability pension^e^^,^^f^	328 108 (6.8)	5563 (3.3)	425 442 (8.7)	15 256 (7.7)	395 336 (8.0)	25 460 (9.8)
Morbidity (1995–1999, 2000–2004, 2005–2009)						
History of inpatient healthcare^g^	836 385 (17.3)	31 349 (18.8)	1 205 971 (24.7)	50 953 (25.9)	1 212 058 (24.7)	67 597 (26.1)
History of suicide attempt^h^	20 780 (0.4)	945 (0.6)	22 450 (0.5)	1185 (0.6)	26 989 (0.5)	1413 (0.5)
Outcome (2000–2003, 2005–2008, 2010–2013)						
Suicide attempt (rate per 100 000 per year^i^)	17 196 (89)	758 (104)	20 902 (107)	910 (106)	20 573 (105)	950 (90)
Suicide (rate per 100 000 per year^i^)	3816 (20)	87 (14)	4942 (25)	127 (16)	3981 (20)	124 (11)
Ratio of suicide attempt and suicide	4.5	7.2	4.2	6.8	5.2	8.0

a Individuals who settled in Sweden as ‘refugee’ or ‘in need of protection’ or ‘humanitarian grounds’.

b Type of residential area: big cities – Stockholm, Gothenburg and Malmö; medium-sized cities – cities with more than 90 000 inhabitants within 30 km distance from the centre of the city; small cities/villages.

c ‘No unemployment’ is the reference category.

d ‘No sickness absence’ is the reference category.

e ‘No disability pension’ is the reference category.

f Individuals having a disability pension during 1999, 2004 and 2009 for respective cohort.

g At least one episode of inpatient healthcare due to certain infectious and parasitic diseases; neoplasms; diabetes mellitus; diseases of the nervous system; diseases of the circulatory system; diseases of the respiratory system; diseases of the digestive system; diseases of the musculoskeletal system and connective tissue; other somatic disorders; depressive disorders; bipolar disorders; anxiety disorders; post-traumatic stress disorders; other stress-related/somatoform disorders and other mental disorders as main or side diagnosis during 1995–1999, 2000–2004 and 2005–2009 for respective cohort

h Any history of suicide attempt for which inpatient healthcare was sought during 1995–1999, 2000–2004 and 2005–2009 for respective cohort.

i Sex- and age-standardised rates.

### Outcome measures

In this study, the outcome measures comprised inpatient healthcare due to suicide attempt and death due to suicide, coded according to ICD-10 codes: X60-X84. Events of undetermined intent (ICD-10 codes: Y10-Y34) were included to limit the potential effects of underreporting and regional and temporal variations in case ascertainment (Linsley *et al*., 2001; Mittendorfer Rutz and Wasserman, 2004; Runeson *et al*., 2015). A sensitivity analysis was performed to check the comparability of the outcome measures, i.e. including or excluding cases with undetermined intent. Analyses with and without these cases yielded the same results (data not shown).

### Statistical analyses

Sex- and age-standardised rates of suicide attempt and suicide for refugees were calculated using Swedish-born as the reference population (Yuan, 2013). The ratios of the rate of suicide attempt and the rate of suicide were also calculated for both Swedish-born and the refugees. Cox proportional hazard regression models were applied to compare refugees in general and from different countries with the Swedish-born population regarding subsequent suicide attempt and suicide. Additionally, sex- and age-stratified regression models were generated (online Supplementary Tables S2 and S3). Results were shown as crude and multivariate hazard ratios (HRs) with 95% confidence intervals (CIs). Covariates were adjusted for in the following manner: Model 1 – socio-demographic covariates; Model 2 – LMM factors and factors in Model 1 and Model 3 – morbidity factors and factors in Model 2. Data were censored in the event of emigration, death due to a cause other than suicide and, end of follow-up, whichever occurred first. The assumption of the proportional hazard was confirmed by plotting log-minus-log Kaplan–Meier survival curves in SPSS version 25. All other analyses were performed using SAS 9.4.

## Ethical statement

Ethical approval was obtained from the Regional Ethical Review Board, Karolinska Institutet, Stockholm, Sweden (review number Dnr: 2007/762–31).

## Results

There was a chronological increase in the number of refugees across the three time periods in the Swedish population as shown in Table 1. Generally, in all three cohorts, the refugees comprised more men and younger individuals relative to the sex and age composition in the Swedish-born population. Refugees had fewer years of education and were more often cohabiting with a partner and children in comparison with the Swedish-born. In all cohorts, almost half of the refugee population was living in big cities compared with a third of the Swedish-born population (Table 1). A higher proportion of refugees received unemployment benefit at baseline in all the cohorts, compared with the Swedish-born population. A lower proportion of refugees than Swedish-born was recipients of sickness absence benefit or disability pension in the 1999 cohort while a higher proportion of refugees than Swedish-born received such benefits in the 2009 cohort (Table 1). Slightly higher proportions with history of inpatient healthcare and almost the same distribution of history of suicide attempt were observed among the refugees and the Swedish-born population in all the cohorts. Sex- and age-standardised suicide attempt rates were lower in refugees, compared with the Swedish-born in the 2009 cohort but higher in the earlier cohorts. Refugees had lower suicide rates (standardised) than Swedish-born in all the cohorts. However, the ratios of suicide attempt rate against the suicide rate were consistently higher in refugees across the three cohorts (Table 1).

### Suicide attempt

During the 2005–2013 follow-up period, refugees, in total, had a lower risk of suicide attempt compared with the Swedish-born in the multivariate analyses (HR 0.82). When stratifying the data by sex and three age groups (16–24, 25–44 and 45–64 years), a lower risk of suicide attempt compared with the Swedish-born population was observed in the fully adjusted models in all strata (HRs ranged from 0.63 to 0. 9) and the lower risks were statistically significant in refugee women 25–44 years, refugee men 16–24 years and refugee men 25–44 years (online Supplementary Table S2). The risk of suicide attempt in refugees from the African region was also lower compared with the Swedish-born population, but no significant differences were observed for refugees from Asia and South America in the adjusted models. However, the risk estimates of suicide attempt among the included Asian countries varied. Refugees from Iraq had a significantly lower risk of suicide attempt (HR 0.85) in the adjusted analysis whilst refugees from Iran had a significantly higher risk of suicide attempt (HR 1.66 and 1.25 in the crude and multivariate models, respectively). The risk of suicide attempt in refugees in comparison with the Swedish-born population, in general and according to their region or country of birth, was marginally explained by socio-demographics but not previous LMM and morbidity factors (Table 2).

**Table 2.** Suicide attempt (first incident) risk during 2005–2013 in refugees from different regions and countries of birth, in comparison with the Swedish-born population, crude and multivariate HRs with 95% CIs

	Population *n* (%)	Suicide attempt, *n* (rate per 100 000 person-years)	Crude HR (CI)	Model 1^a^ HR (CI)	Model 2^b^ HR (CI)	Model 3^c^ HR (CI)
**Swedish-born**	4 886 677 (96.1)	38 279 (89.2)	1	1	1	1
**Refugees**^d^	196 941 (3.9)	1607 (94.2)	1.05 (1.00–1.11)	**0.87 (0.83–0.91)**	**0.83 (0.79–0.87)**	**0.82 (0.78–0.86)**
Non-Western countries^e^						
*Africa* (*region*)	17 966 (0.4)	110 (74.3)	0.83 (0.69–1.00)	**0.52 (0.43–0.63)**	**0.57 (0.47–0.68)**	**0.56 (0.47–0.68)**
Eritrea	2376 (0.1)	10 (48.2)	0.54 (0.29–1.00)	**0.35 (0.19–0.66)**	**0.35 (0.19–0.65)**	**0.38 (0.20–0.70)**
Ethiopia	4367 (0.1)	21 (58.1)	**0.65 (0.42–0.99)**	**0.47 (0.31–0.73)**	**0.46 (0.30–0.71)**	**0.47 (0.31–0.72)**
Somalia	7177 (0.1)	41 (71.9)	0.80 (0.59–1.08)	**0.43 (0.31–0.58)**	**0.50 (0.37–0.68)**	**0.49 (0.36–0.67)**
*Asia* (*region*)	88 489 (1.8)	885 (11.9)	**1.30 (1.21–1.39)**	**1.08 (1.01–1.16)**	1.01 (0.95–1.08)	0.96 (0.90–1.03)
Afghanistan	3112 (0.1)	31 (115.1)	1.29 (0.91–1.83)	1.01 (0.71–1.43)	1.19 (0.84–1.70)	1.25 (0.88–1.77)
Iran	22 822 (0.5)	215 (108.7)	**1.66 (1.49–1.85)**	**1.56 (1.40–1.74)**	**1.36 (1.22–1.51)**	**1.25 (1.12–1.40)**
Iraq	26 065 (0.5)	331 (148.2)	1.09 (0.96–1.23)	0.93 (0.82–1.05)	0.91 (0.80–1.03)	**0.85 (0.75–0.96)**
Syria	30 352 (0.6)	254 (97.1)	1.12 (0.86–1.46)	0.85 (0.65–1.11)	0.77 (0.59–1.00)	0.78 (0.60–1.02)
*South America* (*region*)	10 808 (0.2)	96 (102.3)	1.15 (0.94–1.40)	0.88 (0.72–1.08)	0.83 (0.68–1.01)	0.82 (0.67–1.00)
Chile	8131 (0.2)	81 (114.9)	**1.29 (1.03–1.60)**	0.96 (0.77–1.19)	0.89 (0.71–1.10)	0.89 (0.72–1.11)
Western country^e^						
Former Yugoslavia	68 157 (1.4)	400 (66.5)	**0.75 (0.68–0.82)**	**0.62 (0.57–0.69)**	**0.60 (0.54–0.66)**	**0.61 (0.56–0.68)**

HRs with 95% CIs in bold indicate statistically significant associations (*p* value < 0.05).

The refugee population according to region of birth or country of birth does not add up to 100% because corresponding rows for refugees from other regions (Europe, North America and Oceania) and other countries (other African countries, other Asian countries, other South American countries and other Western countries) are not presented.

a Model 1: adjusted for age, sex, educational level, family situation and type of residential area

b Model 2: adjusted for Model 1 covariates, and LMM factors (unemployment in 2004 (0, 1–180, >180 days)), sickness absence in 2004 (0, 1–90, >90 net days) and disability pension in 2004 (No, Yes)

c Model 3: adjusted for Model 2 covariates, and morbidity factors (main or side diagnosis from inpatient and specialised outpatient healthcare during 2001–2004 for specific somatic or psychiatric disorders, any history of suicide attempt during 2001–2004)

d Individuals who settled in Sweden as ‘refugee’ or ‘in need of protection’ or ‘humanitarian grounds’

e Countries which generated the largest number of refugees to Sweden

### Suicide

Refugees, altogether and when stratified by sex and age groups, had statistically a significant lower risk of suicide in comparison with the Swedish-born population during the 2005–2013 follow-up period, except for refugee females in the age group 16–24 years. They had a non-significant lower risk (Table 3 and online Supplementary Table S3). In general, refugees from different regions and countries of birth also had lower risk estimates of suicide (Table 3). The risk of suicide in refugees from Asia and South America was statistically significantly lower compared with the Swedish-born population, but no differences were observed for refugees from Africa in the multivariate analyses (Table 3). Among the included African countries, only Somalian refugees had a statistically significant lower risk of suicide compared with the Swedish-born population (adjusted HR 0.41). The risk estimates of suicide among the included Asian countries varied considerably. While no differences were observed in the case of refugees from Afghanistan and Iran, refugees from Iraq and Syria had significantly lower HRs for suicide in both crude and multivariate analyses, in comparison with Swedish-born (Table 3). The lowest suicide risk estimate was observed in refugees from Syria (adjusted HR 0.16). The factors in the multivariate analyses only had a marginal effect on the estimates regarding the risk of suicide in refugees.

**Table 3.** Suicide risk during 2005–2013 in refugees from different regions and countries of birth, in comparison with the Swedish-born population, crude and multivariate HRs with 95% CIs

	Population *n* (%)	Suicide, n (rate per 100 000 person-years)	Crude HR (CI)	Model 1^a^HR (CI)	Model 2^b^HR (CI)	Model 3^c^HR (CI)
**Swedish-born**	4 886 677 (96.1)	8918 (20.7)	1	1	1	1
**Refugees**^d^	196 941 (3.9)	226 (13.2)	**0.64 (0.56–0.73)**	**0.64 (0.56–0.73)**	**0.58 (0.51–0.67)**	**0.58 (0.51–0.66)**
Non-Western countries^e^						
*Africa* (*region*)	17 966 (0.4)	27 (18.2)	0.88 (0.60–1.29)	0.71 (0.49–1.04)	0.70 (0.48–1.03)	0.71 (0.48–1.03)
Eritrea	2376 (0.05)	<10^f^	0.93 (0.35–2.48)	0.72 (0.27–1.92)	0.68 (0.26–1.81)	0.72 (0.27–1.91)
Ethiopia	4367 (0.1)	10 (27.6)	1.34 (0.72–2.49)	1.02 (0.55–1.90)	0.97 (0.52–1.81)	0.99 (0.53–1.85)
Somalia	7177 (0.1)	<10^f^	0.51 (0.23–1.13)	**0.40 (0.18–0.88)**	**0.42 (0.19–0.93)**	**0.41 (0.19–0.92)**
*Asia* (*region*)	88 489 (1.8)	104 (13.6)	**0.63 (0.52–0.77)**	**0.59 (0.49–0.73)**	**0.53 (0.43–0.64)**	**0.51 (0.42–0.62)**
Afghanistan	3112 (0.1)	<10^f^	1.07 (0.48–2.39)	1.12 (0.50–2.49)	1.18 (0.53–2.64)	1.20 (0.54–2.68)
Iran	22 822 (0.5)	21 (10.6)	1.12 (0.85–1.47)	1.03 (0.79–1.36)	0.87 (0.66–1.14)	0.82 (0.62–1.08)
Iraq	26 065 (0.5)	52 (23.1)	**0.42 (0.28–0.64)**	**0.41 (0.27–0.61)**	**0.37 (0.25–0.56)**	**0.36 (0.24–0.54)**
Syria	30 352 (0.6)	23 (8.7)	**0.18 (0.04–0.71)**	**0.18 (0.04–0.71)**	**0.16 (0.04–0.63)**	**0.16 (0.04–0.64)**
*South America* (*region*)	10 808 (0.2)	<10^f^	**0.46 (0.24–0.89)**	**0.40 (0.21–0.77)**	**0.38 (0.20–0.72)**	**0.38 (0.20–0.74)**
Chile	8131 (0.2)	<10^b^	0.48 (0.23–1.00)	**0.40 (0.19–0.84)**	**0.37 (0.18–0.77)**	**0.38 (0.18–0.80)**
Western country^e^						
Former Yugoslavia	68 157 (1.4)	64 (10.6)	**0.51 (0.40–0.66)**	**0.59 (0.46–0.76)**	**0.48 (0.34–0.67)**	**0.56 (0.43–0.71)**

HRs with 95% CIs in bold indicate statistically significant associations (*p* value < 0.05).

The refugee population according to region of birth or country of birth does not add up to 100% because corresponding rows for refugees from other regions (Europe, North America and Oceania) and other countries (other African countries, other Asian countries, other South American countries and other Western countries) are not presented.

a Model 1: adjusted for age, sex, educational level, family situation and type of residential area.

b Model 2: adjusted for Model 1 covariates and LMM factors (unemployment in 2004 (0, 1–180, >180 days)), sickness absence in 2004 (0, 1–90, >90 net days) and disability pension in 2004 (No, Yes).

c Model 3: adjusted for Model 2 covariates and morbidity factors (main or side diagnosis from inpatient and specialised outpatient healthcare during 2001–2004 for specific somatic or psychiatric disorders, any history of suicide attempt during 2001–2004).

d Individuals who settled in Sweden as ‘refugee’ or ‘in need of protection’ or ‘humanitarian grounds’.

e Countries which generated the largest number of refugees to Sweden.

f For ethical reasons i.e. the risk of identification of individuals, if the number of suicides is <10, it is not reported.

### Time period effects

The suicide attempt and suicide rates per 100 000 person-years for refugees tended to decline across the 1999, 2004 and 2009 cohorts, particularly for suicide attempt. Compared with the Swedish-born, refugees had a non-significantly higher risk of suicide attempt in the 1999 cohort (adjusted HR: 1.05) but a lower risk of suicide attempt in the 2004 and 2009 cohort (adjusted HRs: 0.89 and 0.80 respectively). The adjusted HRs for suicide were 0.63, 0.61 and 0.56 in the first, second and the last cohorts, respectively (Table 4).

**Table 4.** Crude and multivariate HRs with 95% CIs for suicide attempt and suicide in refugees, compared with the Swedish-born population, in three time period cohorts i.e. 1999, 2004 and 2009 cohort, each with 4 years of follow-up

Cohort	Refugee status	Person-years	*n* (rate per 100 000 person-years)	Crude HR (CI)	Model 1^a^ HR (CI)	Model 2^b^ HR (CI)	Model 3^c^ HR (CI)
Suicide attempt							
1999	Swedish-born	19 123 265	17 196 (89.9)	1	1	1	1
	Refugees^d^	657 915	758 (114.8)	**1.28 (1.19–1.38)**	1.06 (0.98–1.14)	1.07 (0.99–1.15)	1.05 (0.97–1.13)
2004	Swedish-born	19 346 957	20 902 (108.0)	1	1	1	1
	Refugees^d^	775 521	910 (116.7)	**1.09 (1.02–1.16)**	**0.91 (0.85–0.97)**	**0.88 (0.82–0.94)**	**0.89 (0.83–0.95)**
2009	Swedish-born	19 453 710	20 573 (105.8)	1	1	1	1
	Refugees^d^	1 019 002	950 (93.1)	**0.88 (0.83–0.94)**	**0.76 (0.71–0.81)**	**0.75 (0.70–0.80)**	**0.80 (0.75–0.86)**
Suicide							
1999	Swedish-born	19 157 913	3816 (19.9)	1	1	1	1
	Refugees^d^	659 427	87 (13.2)	**0.66 (0.54–0.82)**	**0.68 (0.55–0.84)**	**0.63 (0.51–0.78)**	**0.63 (0.51–0.78)**
2004	Swedish-born	19 389 665	4942 (25.5)	1	1	1	1
	Refugees^d^	777 448	127 (16.3)	**0.64 (0.54–0.77)**	**0.67 (0.56–0.79)**	**0.61 (0.51–0.72)**	**0.61 (0.51–0.73)**
2009	Swedish-born	19 496 913	3981 (20.4)	1	1	1	1
	Refugees^d^	1 021 079	124 (12.2)	**0.60 (0.50–0.71)**	**0.59 (0.49–0.71)**	**0.53 (0.45–0.64)**	**0.56 (0.46–0.67)**

HRs with 95% CIs in bold indicate statistically significant associations (*p* value < 0.05).

a Model 1: adjusted for age, sex, educational level, family situation and type of residential area.

b Model 2: adjusted for Model 1 covariates and LMM factors (unemployment in 1999, 2004 and 2009 (0, 1–180, >180 days)), sickness absence in 1999, 2004 and 2009 (0, 1–90, >90 net days) and disability pension in 1999, 2004 and 2009 (No, Yes) for respective cohort.

c Model 3: adjusted for Model 2 covariates and morbidity factors (main or side diagnosis from inpatient healthcare during 1995–1999, 2000–2004 and 2005–2009 for specific somatic or psychiatric disorders, any history of suicide attempt during 1995–1999, 2000–2004 and 2005–2009 for respective cohort).

d Individuals who settled in Sweden as ‘'refugee’ or ‘in need of protection’ or ‘humanitarian grounds’.

## Discussion

### Main findings

Compared with the Swedish-born population, almost all refugee groups, according to their country of birth, had a lower risk of suicide attempt and suicide during a 9-year follow-up period. The risk of suicide attempt in refugees from Asian and South American countries was mostly non-significant, but higher in the case of Iran, compared with the Swedish-born. Risk estimates for suicide attempt decreased slightly across time. A number of covariates including socio-demographics, previous LMM and morbidity factors only marginally influenced the associations between refugee status and subsequent suicide attempt and suicide.

The risk of suicide attempt and suicide was found to be lower, compared with the Swedish-born population, in the majority of the refugee groups in our study. Several factors may have contributed to these findings. First, the ‘healthy migrant effect’ may hold true for refugees (Norredam *et al*., 2013). A health selection bias might have occurred for the refugees who were able to overcome the pre- and peri-migratory difficult circumstances to reach the new country. Second, our refugee population had already received residence permits in Sweden at inclusion and asylum seekers were not part of our cohorts. Previously, higher rates of suicidal behaviour in asylum seekers, compared with the Dutch population were reported (Goosen *et al*., 2011). The difference between our results and these findings could partly be due to the stressors related to the asylum process, which might have been overcome in the refugee groups who were already residents in Sweden (Goosen *et al*., 2011). Moreover, around 85% of the refugees in all the cohorts in this study had been residing in Sweden for longer than 5 years (data not shown). The long duration of residence in the host country may have helped them to overcome some of the most acute difficulties associated with acculturation and build up family and community connections, that in turn might have worked in a protective way against suicidal behaviour in these longer established refugee groups.

Moreover, since most of the refugee groups were from Muslim majority countries, alcohol consumption, an important potential confounder in this context that has been related to suicide, could be lower in this refugee population than in the Swedish-born population (Amundsen, 2012), which may partly explain the lower risk of suicidal behaviour in refugees. Finally, cultural differences in the experience, expression and stigmatisation of mental disorders, in addition to the differences in culture and religion-driven attitudes towards suicidal behaviour in refugees and the Swedish-born population could also have influenced our results.

### Risk of suicide attempt

We found a significantly lower risk of suicide attempt in refugees compared with the Swedish-born population. The risk of suicide attempt was significantly lower in refugees from Eritrea, Ethiopia, Somalia and former Yugoslavia, compared with the Swedish-born population. Although there are no previous studies on this research question available for comparison, lower suicide attempt rates, similar to our findings, have been reported earlier for migrants (not distinguishing between refugees and non-refugee migrants) from former Yugoslavia and Ethiopia (Westman *et al*., 2003; Bursztein Lipsicas *et al*., 2012).

One notable finding in our study is that, compared with the Swedish-born, refugees from Iran had higher risk estimates of suicide attempt in the adjusted analyses (Table 2). Previously, a study reported significantly higher age-adjusted HRs of suicide attempt for migrants from Iran compared with the Swedish-born comparison group (Westman *et al*., 2003). A majority of the refugees from Iran in Sweden were reported to be more secular and more educated than the general population in Iran (Lewin, 2001). Factors related to this apparent heightened risk for suicide attempts compared with the Swedish-born should be further explored.

### Risk of suicide

Our results showed lower risk estimates of suicide in refugees, altogether and stratified by sex and age groups, with some variation across country of birth. Because of the lack of previous studies that investigated the risk of suicide in refugees from specific countries of birth, these findings are not directly comparable with any previous results. However, a lower risk of suicide for male but not for female refugees, compared with their native Danish counterparts, reported by Norredam *et al*., 2013 also partially agrees with our findings. Moreover, previous studies reported a lower risk of suicide in immigrant subgroups according to their region of birth, compared with the population in the host country (Di Thiene *et al*., 2015; Puzo *et al*., 2017). Our results can be considered to be in line with these findings because the migrant groups from Africa and the Middle East in these studies were mainly comprised people from the main refugee-generating countries to Sweden.

Although, in comparison with the Swedish-born, we found a lower risk of suicide in most refugee groups according to their country of birth, the ratios of the suicide attempt rate over the suicide rate were consistently higher in refugees across all the time period cohorts. This may reflect a higher stigmatisation of suicide than suicide attempt in refugees compared with the Swedish-born. Moreover, the choice of more lethal suicide methods in the Swedish-born may also contribute to this finding. Future studies are required to investigate such associations.

### Time period effects

Our results indicated minor time period effects in the association of refugee status and subsequent suicide attempt – the risk estimate decreased from 1999 through 2004 to 2009 cohorts. This suggests that changes in health care management, social insurance regulations and migration policies might have no major influence in the association between refugee status and subsequent suicide attempt. Therefore, national level policy changes regarding mental healthcare and social insurance systems that happened in Sweden during the aforementioned periods are unlikely in themselves to account for the risk of suicidal behaviour in refugees.

### Influence of covariates

Socio-demographic, previous LMM and morbidity factors had only a marginal effect on the estimates in the adjusted models. This is surprising due to the observed differences in these covariates between refugees and the Swedish-born population. While the possibility of residual confounding in the measurement of the covariates cannot be fully disregarded, it seems likely that the reduced risk of suicide attempt and suicide among refugees is driven by other factors than the covariates mentioned. Therefore, more knowledge on risk and particularly also protective factors of suicidal behaviour in refugees is warranted.

### Strengths and limitations

The use of high-quality nationwide register data (Brooke *et al*., 2017) and using a prospective cohort design are strengths of this study. This allowed us to follow the whole population of Sweden at different time points regarding rare outcome measures like suicide attempt and suicide. We could also stratify the analyses according to the specific countries of birth of refugees. Additionally, the possibility to adjust for an array of socio-demographic, previous LMM and morbidity confounders is another strength of this study.

There are also limitations to be considered. We included only the suicide attempts registered in inpatient healthcare. Previously, among suicide attempters, only 25% were estimated to have received hospitalised care (Krug *et al*., 2002). Therefore, we may not draw conclusions on less severe forms of suicide attempts. Also, reliance on hospital-based sources means that the underreporting of suicide attempts could have been differential in this study. For example, due to religious and cultural stigma, suicide attempts might be more underreported in refugees than in the Swedish-born population. However, the inclusion of events of undetermined intent (ICD-10 codes: Y10–Y34) in our study may have, to some extent, reduced this sort of reporting bias. Second, the refugee population in this study consisted also of individuals, who were granted residence permit on ‘in need of protection’ and ‘humanitarian grounds’. Generally, morbidity in the latter group is considered to be more than any other group of people who get residence permits in Sweden (Hollander *et al*., 2011). This might have introduced some negative mental health selection in the refugee group in our study. However, our sensitivity analyses, including and excluding these individuals, revealed no significant differences in the results (data not shown). Additionally, there might be some residual confounding in our analyses regarding the measurement of the covariates. For example, specialised healthcare covers only the most severe forms of morbidity and refugees might be underrepresented due to lower healthcare seeking and differences in access to specialised healthcare (Sundvall *et al*., 2015). Also, register data regarding morbidity factors could be less complete in refugees, compared with the Swedish-born population, because of their shorter duration of residence in Sweden. Moreover, we could not adjust for some potential confounders in the analyses of the risk of suicide attempt and suicide, e.g. alcohol consumption, due to the lack of data. There might be differences in rates of suicidal behaviour in refugees compared with individuals in their respective countries of birth. Still, the scarceness of studies and low quality of data in most of the countries of birth of refugees make a consistent comparison of rates of suicidal behaviour impossible. Finally, our findings are probably not generalisable to other settings such as refugee camps, asylum seeking populations, refugees to low or middle income countries or countries with stricter regulations regarding immigration.

## Conclusions

Despite reported higher rates of mental disorders in refugees in previous studies, we found a lower risk of suicide attempt and suicide in refugees compared with the Swedish-born population. The risk of suicide attempt in refugees decreased somewhat with time. The association between the refugee status and subsequent suicidal behaviour was not explained by a number of confounders. Future studies are warranted to increase our knowledge regarding both risk and protective factors for suicide attempt and suicide in refugee populations.

## Supplementary material

For supplementary material accompanying this paper visit https://doi.org/10.1017/S0033291719003167. click here to view supplementary material
