# Supplementary material for: Suicide attempt and suicide in refugees in Sweden – a nationwide population-based cohort study
Source: Psychol Med. 2019 Dec 20;51(2):254–63. doi: 10.1017/S0033291719003167 (PMC7893509; doi:10.1017/S0033291719003167)
Supplement: Supplementary file 1 [file S0033291719003167sup001.docx]

| Supplementary Table S1: List of specific psychiatric and somatic disorders used to create the covariates on history of inpatient or specialised outpatient healthcare | | | |
| --- | --- | --- | --- |
| **Variable** | **Description** | **Categories** | **Comments** |
| 1. History of inpatient healthcare | Having at least one inpatient healthcare due to any of the following somatic or psychiatric diagnoses according to the corresponding International Classification of Diseases version 10 (ICD-10) codes: certain infectious and parasitic diseases (A00-B99); neoplasms (C00-D48); diabetes mellitus (E10-E14), diseases of the nervous system (G00-G99); diseases of the circulatory system (I00-I99); diseases of the respiratory system (J00-J99); diseases of the digestive system (K00-K93); diseases of the musculoskeletal system and connective tissue (M00-M99); other somatic disorders (E00-E09, E15-E90, H00-H95, L00-99, N00-T99), depressive disorders (F32-F34), bipolar diseases (F30-F31) anxiety disorders (F40-F42, F44-F48), PTSD (F43.1), other stress-related /somatoform disorders (F38-F39, F43.0, F43.2-F43.9) and other mental disorders (F01-F29, F50-F99) | 1. No history of inpatient healthcare 2. History of inpatient healthcare | - Included in the analyses of 1999, 2004 and 2009 cohort with four years of follow-up. - Measured for five years preceding the start of the follow-up of each cohort (1995-1999, 2000-2004, 2005-2009 for the 1999, 2004 and 2009 cohort respectively. |
| 1. History of inpatient or specialised outpatient healthcare | Having at least one inpatient or specialised outpatient healthcare due to any of the following somatic or psychiatric diagnoses according to the corresponding International Classification of Diseases version 10 (ICD-10) codes: certain infectious and parasitic diseases (A00-B99); neoplasms (C00-D48); diabetes mellitus (E10-E14), diseases of the nervous system (G00-G99); diseases of the circulatory system (I00-I99); diseases of the respiratory system (J00-J99); diseases of the digestive system (K00-K93); diseases of the musculoskeletal system and connective tissue (M00-M99); other somatic disorders (E00-E09, E15-E90, H00-H95, L00-99, N00-T99), depressive disorders (F32-F34), bipolar diseases (F30-F31) anxiety disorders (F40-F42, F44-F48), PTSD (F43.1), other stress-related /somatoform disorders (F38-F39, F43.0, F43.2-F43.9) and other mental disorders (F01-F29, F50-F99) | 1. No history of inpatient or specialised outpatient healthcare 2. History of inpatient or specialised outpatient healthcare | - Included in the analyses of the 2004 cohort with nine years of follow-up. - Measured for four years (2001-2004) preceding the start of the follow-up. |

| Supplementary Table S2: Suicide attempt risk during 2005-2013 in refugees**^†^**, stratified by sex and age groups, in comparison with the Swedish-born population belonging to the same sex and age group, crude and multivariate hazard ratios (HRs) with 95% confidence intervals (CIs) | | | | | | |
| --- | --- | --- | --- | --- | --- | --- |
|  | **Person-years** | **Suicide, n (rate per  100,000 person-years)** | **Crude HR (CI)** | **Model 1^‡^ HR (CI)** | **Model 2^§^ HR (CI)** | **Model 3^¤^ HR (CI)** |
| **Women** |  |  |  |  |  |  |
| Swedish-born, 16-24 years | 3,601,431 | 6,868 (190.7) | 1 | 1 | 1 | 1 |
| Refugees, 16-24 years | 170,045 | 345 (202.9) | 1.06 (0.95-1.18) | **0.84 (0.75-0.93)** | **0.89 (0.79-0.99)** | 0.90 (0.81-1.01) |
| Swedish-born, 25-44 years | 8,679,053 | 8,471 (97.6) | 1 | 1 | 1 | 1 |
| Refugees, 25-44 years | 362,464 | 384 (105.9) | 1.08 (0.98-1.20) | **0.78 (0.70-0.87)** | **0.87 (0.79-0.97)** | **0.86 (0.78-0.96)** |
| Swedish-born, 45-64 years | 8,740,106 | 5,392 (61.7) | 1 | 1 | 1 | 1 |
| Refugees, 45-64 years | 194,213 | 124 (63.8) | 1.04 (0.87-1.24) | 0.94 (0.79-1.13) | 0.88 (0.73-1.05) | 0.84 (0.70-1.01) |
| **Men** |  |  |  |  |  |  |
| Swedish-born, 16-24 years | 3,837,924 | 4,898 (127.6) | 1 | 1 | 1 | 1 |
| Refugees, 16-24 years | 195,211 | 219 (112.2) | 0.88 (0.77-1.01) | **0.64 (0.56-0.74)** | **0.62 (0.54-0.72)** | **0.63 (0.55-0.73)** |
| Swedish-born, 25-44 years | 9,161,618 | 7,379 (80.5) | 1 | 1 | 1 | 1 |
| Refugees, 25-44 years | 515,820 | 393 (76.2) | 0.94 (0.85-1.05) | **0.84 (0.76-0.93)** | **0.72 (0.65-0.80)** | **0.74 (0.67-0.83)** |
| Swedish-born, 45-64 years | 8,872,659 | 5,271 (59.4) | 1 | 1 | 1 | 1 |
| Refugees, 45-64 years | 267,441 | 142 (53.1) | 0.89 (0.76-1.06) | 1.05 (0.89-1.24) | 0.85 (0.72-1.01) | 0.84 (0.71-1.00) |
| ^†^ Individuals who settled in Sweden as 'refugee' or 'in need of protection' or 'humanitarian grounds'.  ^‡^ Model 1: adjusted for educational level, family situation, type of residential area.  ^§^ Model 2: adjusted for Model 1 covariates and labour market marginalisation factors (unemployment in 2004 (0, 1-180, >180 days), sickness absence in 2004 (0, 1-90, >90 net days) and disability pension in 2004 (No, Yes).  ^¤^ Model 3: adjusted for Model 2 covariates and morbidity factors (main or side diagnosis from inpatient and specialised outpatient healthcare during 2001-2004 for specific somatic or psychiatric disorders, any history of suicide attempt during 2001-2004).  ^Ʊ^ Countries which generated the largest number of refugees to Sweden.  HRs with 95% CIs in bold indicate statistically significant associations (p-Value <0.05) | | | | | | |

| Supplementary Table S3: Suicide risk during 2005-2013 in refugees**^†^**, stratified by sex and age groups, in comparison with the Swedish-born population belonging to the same sex and age group, crude and multivariate hazard ratios (HRs) with 95% confidence intervals (CIs) | | | | | | |
| --- | --- | --- | --- | --- | --- | --- |
|  | **Person-years** | **Suicide, n (rate per  100,000 person-years)** | **Crude HR (CI)** | **Model 1^‡^ HR (CI)** | **Model 2^§^ HR (CI)** | **Model 3^¤^ HR (CI)** |
| **Women** |  |  |  |  |  |  |
| Swedish-born, 16-24 years | 3,638,631 | 343 (9.4) | 1 | 1 | 1 | 1 |
| Refugees, 16-24 years | 171,910 | 12 (7.0) | 0.74 (0.42-1.32) | 0.60 (0.33-1.08) | 0.67 (0.37-1.19) | 0.68 (0.38-1.21) |
| Swedish-born, 25-44 years | 8,720,229 | 996 (11.4) | 1 | 1 | 1 | 1 |
| Refugees, 25-44 years | 364,479 | 23 (6.3) | **0.55 (0.37-0.84)** | **0.45 (0.30-0.68)** | **0.53 (0.35-0.81)** | **0.53 (0.35-0.81)** |
| Swedish-born, 45-64 years | 8,764,942 | 1,242 (14.2) | 1 | 1 | 1 | 1 |
| Refugees, 45-64 years | 194,802 | 17 (8.7) | **0.62 (0.38-0.99)** | 0.66 (0.41-1.08) | **0.61 (0.38-0.99)** | **0.60 (0.37-0.98)** |
| **Men** |  |  |  |  |  |  |
| Swedish-born, 16-24 years | 3,860,423 | 988 (25.6) | 1 | 1 | 1 | 1 |
| Refugees, 16-24 years | 196,271 | 28 (14.3) | **0.56 (0.38-0.81)** | **0.47 (0.32-0.68)** | **0.45 (0.31-0.66)** | **0.46 (0.31-0.66)** |
| Swedish-born, 25-44 years | 9,194,606 | 2,344 (25.5) | 1 | 1 | 1 | 1 |
| Refugees, 25-44 years | 517,607 | 93 (18.0) | **0.71 (0.57-0.87)** | **0.70 (0.56-0.86)** | **0.61 (0.49-0.75)** | **0.62 (0.50-0.77)** |
| Swedish-born, 45-64 years | 8,894,464 | 3,005 (33.8) | 1 | 1 | 1 | 1 |
| Refugees, 45-64 years | 268,048 | 53 (19.8) | **0.59 (0.45-0.77)** | **0.69 (0.52-0.91)** | **0.60 (0.45-0.78)** | **0.60 (0.45-0.78)** |
| ^†^ Individuals who settled in Sweden as 'refugee' or 'in need of protection' or 'humanitarian grounds'.  ^‡^ Model 1: adjusted for educational level, family situation, type of residential area.  ^§^ Model 2: adjusted for Model 1 covariates and labour market marginalisation factors (unemployment in 2004 (0, 1-180, >180 days), sickness absence in 2004 (0, 1-90, >90 net days) and disability pension in 2004 (No, Yes).  ^¤^ Model 3: adjusted for Model 2 covariates and morbidity factors (main or side diagnosis from inpatient and specialised outpatient healthcare during 2001-2004 for specific somatic or psychiatric disorders, any history of suicide attempt during 2001-2004).  ^Ʊ^ Countries which generated the largest number of refugees to Sweden.  HRs with 95% CIs in bold indicate statistically significant associations (p-Value <0.05) | | | | | | |
